# VAWCM-Instillation Improves Delayed Primary Fascial Closure of Open Septic Abdomen

**DOI:** 10.1155/2014/245182

**Published:** 2014-12-07

**Authors:** Qingsong Tao, Jianan Ren, Zhenling Ji, Shengli Liu, Baochai Wang, Yu Zheng, Guosheng Gu, Xinbo Wang, Jieshou Li

**Affiliations:** ^1^Department of General Surgery, Affiliated Zhongda Hospital, Southeast University Medical School, 87 Dingjiaqiao, Nanjing, Jiangsu 210089, China; ^2^Research Institute of General Surgery, Jinling Hospital, Nanjing University Medical School, 305 East Zhongshan Road, Nanjing, Jiangsu 210093, China; ^3^Department of Surgery, Haian People's Hospital, Nantong University Medical School, 17 Zhongba Road, Nantong, Jiangsu 226600, China

## Abstract

*Background.* Failure to achieve delayed primary fascial closure (DPFC) is one of the main complications of open abdomen (OA), certainly when abdominal sepsis is present. This retrospective cohort study aims to evaluate the effect of combined therapy of vacuum-assisted mesh-mediated fascial traction and topical instillation (VAWCM-instillation) on DPFC in the open septic abdomen. *Methods.* The patients with abdominal sepsis who underwent OA using VAWCM were included and divided into the instillation and noninstillation (control) groups. The DPFC rate and other outcomes were compared between the two groups. *Results.* Between 2007 and 2013, 73 patients with open septic abdomen were treated with VAWCM-instillation and 61 cases with VAWCM-only. The DPFC rate in the instillation group was significantly increased (63% versus 41%, *P* = 0.011). The mortality with OA was similar (24.6% versus 23%, *P* = 0.817) between the two groups. However, time to DPFC (*P* = 0.003) and length of stay in hospital (*P* = 0.022) of the survivals were significantly decreased in the instillation group. In addition, VAWCM-instillation (OR 1.453, 95% CI 1.222–4.927, *P* = 0.011) was an independent influencing factor related to successful DPFC. *Conclusions.* VAWCM-instillation could improve the DPFC rate but could not decrease the mortality in the patients with open septic abdomen.

## 1. Introduction

Open abdomen (OA) has been considered a common technique in the management of abdominal compartment syndrome (ACS), severely abdominal trauma, and abdominal sepsis [1, 2]. Comparing to the trauma patients, a longer time may be required to achieve delayed primary fascial closure (DPFC) in the patients with open abdominal sepsis [3]. If DPFC could not be achieved, skin-only closure or split-thickness skin grafting would usually be applied after wound infection was cleared and healthy granulation bed was formed, and the planned ventral hernia has to be dealt with later [4].

The role of OA in the management of abdominal sepsis has been a controversial issue. Some studies failed to show any significant benefit for the open septic abdomen using traditional passive abdominal packing [5, 6], which was unable to drain effectively any toxic or infected intra-abdominal fluid. Recent experimental and clinical studies have indicated that vacuum-assisted closure (VAC) is associated with superior outcomes in the treatment of open abdominal sepsis [7–9]. Vacuum-assisted wound closure and mesh-mediated fascial traction (VAWCM) seemed to be a promising technique providing a higher DPFC rate and few complications after long-term treatment of OA [10–12]. It has become a routine temporary abdominal closure (TAC) technique for open septic abdomen at our institutions [13].

Recently, VAC-instillation therapy (KCI, San Antonio, USA), a combined technique of VAC or negative pressure wound therapy (NPWT) and topical instillation, has been introduced in wound care. Studies have demonstrated that VAC-instillation showed a significant decrease in the mean time of bioburden reduction, wound closure, and hospital discharge compared with VAC-only method [14–16]. D'Hondt et al. reported the encouraging results of the VAC-instillation in five patients with open septic abdomen [17, 18]. However, no clinical data have been attempted to evaluate the role of VAWCM-instillation in the management of open septic abdomen. The main purpose of this study was to evaluate the role of VAWCM-instillation in the management of open septic abdomen.

## 2. Patients and Methods

### 2.1. Study Design and Patients

This was a multiple-center retrospective cohort study on the patients with abdominal sepsis who experienced open abdomen (OA) using VAWCM between January 2007 and November 2013. The patients with septic OA of grades 1–3, as classified by Björck et al. [19], were suitable for inclusion. Exclusion criteria included < 18 years, frozen OA with adherent bowel (grade 4), preexisting large ventral hernia, end-stage renal disease, severe liver disease, uncontrolled diabetes, critical wound ischemia, and any issue with an obviously high risk of delayed wound healing. Conventional therapies [20] for patient-specific sepsis were also recorded for analyses. Delayed primary fascial closure (DPFC) referred to the ability to achieve fascial closure during the initial hospital stay [11].

### 2.2. VAWCM and Topical Instillation

**Figure 1:**
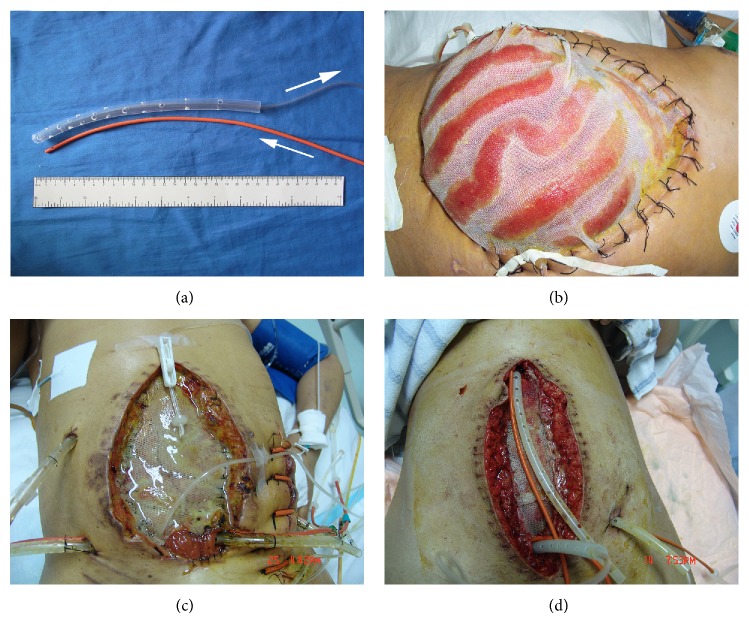
(a) Construction of the VAC-instillation tubes with washing tube (red) and suction “sleeve” tubes. Arrows indicate the destination of washing solution flow. Outer tube with 10–15 side holes 1 cm from the blind distal end. ((b)–(d)) Representative OA cases treated with topical instillation and VAWCM. The topical solution (saline) flowed across the open abdomen to 300–500 mL/h, to clean and flush the abdomen. The abdominal fluid was captured by the VAC system.

The principle of VAWCM has been described previously [11]. In brief, a sterile nonadhesive plastic sheet was placed intra-abdominally to cover the viscera and an oval-shaped polypropylene mesh (Prolene; Ethicon, Johnson & Johnson, Somerville, NJ) was sutured to the fascial edges with a running 0 monofilament suture. Moist gauze dressings were placed overlying these drapes. Then, as shown in Figure 1, instillation and suction catheters (instillation) or only suction catheters (control) were embedded in gauze dressings. The suction catheter of sump drain was attached to an aspiration pump with continuous topical negative pressure of 150 to 200 millibars. Persistent instillation by normal saline (150–300 mL/h) was performed, meantime, through the instillation catheter. The drains were covered with a layer of dry laparotomy pads and the wound was sealed with adhesive plastic dressings. This TAC system was changed every 2 to 3 days with debridement as needed, followed by catheter replacing and gauze redressing. Meanwhile, the possibility to close the abdomen was evaluated. If possible, the abdominal wall was closed. Otherwise, the mesh was cut in the midline and tightened by suturing in the midline with a running 0 monofilament suture, keeping the viscera from protruding and putting some tension on the abdominal wall. When 3–5 cm of separation of the fascial edges remained with weak tension, fascial closure was considered followed by skin closure or split skin grafting.

### 2.3. Other Strategies for the Management of Open Septic Abdomen

The early postoperative management after OA was to correct the oxygen and energy debt, hypothermia, and coagulopathy [21]. Sedation [22], analgesia [22], and/or neuromuscular blockade [23] were used to decrease intra-abdominal pressure. Although early goal-directed fluid resuscitation is critical to correct hypovolemia, restricted crystalloid fluid infusion or negative fluid balance was paid more attention to avoid the risk of worsening intra-abdominal hypertension. Early enteral feeding (EEF), defined as a successful initiation of enteral feeding within 1 week, was recommended for the patients without any intolerance response [24, 25]. Antibiotic coverage would initially be broad to cover the wide range of skin and bowel flora and then tapered according to intraoperative culture results [22].

### 2.4. Outcome Measurement

For each patient, the daily flow charts with clinical data were reviewed during OA treatment. Mortality, fascial closure, length of stay in hospital, and postoperative complications were collected.

### 2.5. Statistical Analysis

Data are expressed as mean ± SD. Student's *t*-test was performed for continuous parametric variables, Mann-Whitney *U* test for continuous nonparametric variables, and Chi-square test or Fisher's exact test for categorical variables. Fascial closure rates were analyzed by the Kaplan-Meier method and compared by log-rank test. Multivariable analysis of factors influencing successful delayed primary fascial closure was performed using logistic regression, with results being presented as odds ratio (OR) with 95% confidence interval (CI). All data were analyzed using SPSS V20 (IBM, Armonk, New York, USA). Statistical significance was set at *P* < 0.05.

## 3. Results

### 3.1. Patients

**Figure 2:**
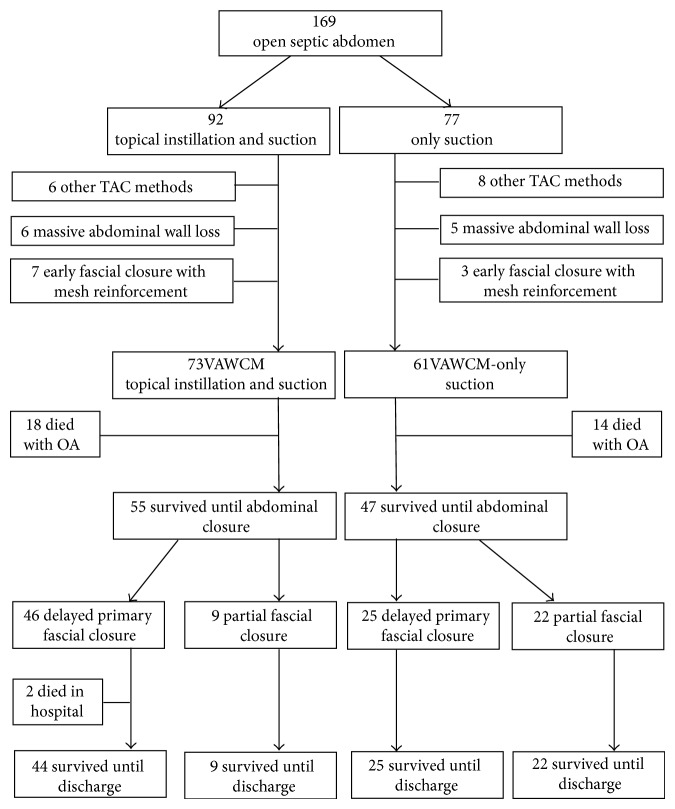
Flowchart describing the delayed primary fascial closure and mortality included in this study.

As shown in Figure 2, 169 (116 males and 53 females) consecutive patients with open septic abdomen were included between January 2007 and November 2013, 92 in instillation group and 77 in control group. 10 patients underwent early fascial closure at the time of first dressing change (less than 5 days, 7 in the instillation group and 3 in the control group, *P* = 0.349), 14 patients were treated with other TAC methods (3 with skin only, 3 with Dacron strip, and 8 with Bogota bag; 6 in the instillation group and 8 in the control group, *P* = 0.364), and 11 patients with massive abdominal wall loss were also excluded (6 in the instillation group and 5 in the control group, *P* = 0.994), without being further considered, leaving 134 patients (92 males and 42 females, 73 in instillation group and 61 in control group) in the final analysis. Patient characteristics are summarized in Table 1.

### 3.2. Mortality and Postoperative Complications

Thirty-two patients (23.9%) died before abdominal closure, 18 in the instillation group and 14 in the control group (*P* = 0.817, Table 2). Infectious complications were found in almost all the patients in whom primary fascial closure was unsuccessful or who died before abdominal closure (61/63, 96.8%), such as postoperative intra-abdominal abscess (31/63, 49.2%) or complicated fistula formation (27/63, 42.8%) (Table 2). Of note, topical instillation decreased the secondary intra-abdominal abscess after OA (16.4% versus 31.1%, *P* = 0.044).

### 3.3. DPFC Rate

Delayed primary fascial closure was achieved in 46 patients (63%) in the instillation group, significantly higher (*P* = 0.011, Table 2) than the control group (25 patients, 41%). In all the survivals with abdominal closure, delayed primary fascial closure was achieved in 83.6% (46 of 55 patients) in the instillation group, significantly higher than 53.2% (25 of 47) in the control (*P* = 0.001, Table 2).

### 3.4. Length of Stay in Hospital

**Figure 3:**
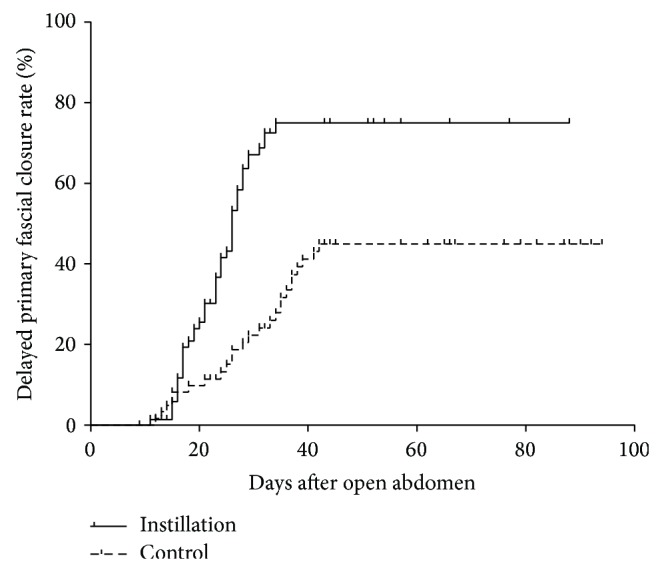
Delayed primary fascial closure rates in the two groups. The curves were calculated by Kaplan-Meier method and compared by log-rank test (*P* = 0.013).

The average length of stay (LOS) in hospital was 39 ± 15 d (19–88) and 44 ± 19 d (27–79) in the groups with and without topical instillation, respectively (*P* = 0.022). Moreover, in the patients who achieved fascial closure, it took less time for the instillation group (23 ± 7 days) than the control (28 ± 10 days, *P* = 0.003). As shown in Figure 3, the topical instillation technique significantly improved the fascial closure rate, as compared with the control (*P* = 0.013).

### 3.5. Factors Related to DPFC

All data were pooled to analyze the confounding factors that could influence the delayed primary fascial closure rate (Table 3). Independent factors of DPFC were identified by multivariable logistic regression analysis. The topical instillation was an only significant positive risk factor for DPFC (OR = 2.453, 95% CI 1.222–4.927, *P* = 0.011). Other independent factors might positively influence the DPFC rate but, without significance, were restricted crystalloid fluid infusion (OR = 1.793, 95% CI 0.900–3.569; *P* = 0.095) and early enteral feeding (OR = 1.670, 95% CI 0.827–3.372; *P* = 0.113).

## 4. Discussion

Open abdomen has become an important approach for damage-control procedures, ACS, and severe intra-abdominal sepsis. However, when delayed primary fascial closure cannot be achieved, OA can be associated with serious complications including giant hernia formation, wound infection, and intestinal fistula [26]. According to recent studies, the VAC method has the best results for patients with open septic abdomen, as compared with other TAC methods [27]. The documented benefits of VAC are enhanced tissue perfusion, promotion of granulation, increased bacterial wound clearance, and decreased tissue edema [28]. The VAWCM and continuous instillation could provide the effective drainage by reducing the occlusion of suction tube, enable effective debridement by diluting infected/necrotized tissues, and decrease the incidence of fistula by providing relatively moist ambient [29].

Early enteral feeding (EEF) would be advocated after open abdomen [30]. EEF is associated with increased primary fascial closure and decreased intestinal fistula, infectious complications, ICU stays, and hospital costs [24, 25]. Cothren et al. [31] verified that tube feeding did not increase the risk of ACS in the recently closed OA. In our study, 48 of 83 (58%) patients who received EEF experienced successful DPFC, while 23 patients (45%) underwent successful DPFC in 51 patients who did not receive EEF. A controlled prospective study with larger sample size may be required to further evaluate the role of EEF in such population.

In the past few years, concept and approach of fluid resuscitation have changed, including a more balanced transfusion protocol (including 1 : 1 ratios), limited use of crystalloid resuscitation, and widespread use of damage-control approaches [32]. Aggressive crystalloid infusion can lead to fluid volume overload and increased risks of ACS, pulmonary edema, and acute respiratory distress syndrome [33]. Restrictive crystalloid fluid infusion (RCFI) may decrease the incidence of ACS and increase early closure rates of OA [34, 35]. In this study, our results indicated that DPFC was achieved in 44 of 74 (59%) patients with RCFI and 27 of 60 (45%) patients without RCFI.

One of the challenges of the study is complicated postoperative fistula. This embarrassing issue would cause DPFC to be almost impossible. Diaz Jr. et al. [36] reported complicated fistula formed in as many as 75% of patients if a nonadherent barrier or omentum was not placed over the viscera for protection. In this study, nonadhesive plastic drapes, which were also filled with sterile vaseline, were placed between viscera and mesh. However, complicated postoperative fistula still occurred in 27 (20%) patients.

In this study, unexpectedly, the mortality rate under the instillation method was not significantly different in comparison with the control approach. The data indicated that the main causes of death are the continuum of clinical events from severe sepsis to septic shock and to multiple organ dysfunction syndromes (MODS). The mortality was not related to the application of instillation method in this study. But further prospective studies are required.

There are some limitations of this study. The length of ICU stay did not account for a clinical outcome in our study. The hospitals have not been expanded before the government increased the financial investment in the medical service 5-6 years ago. Even now, we have not offered enough regular beds yet for our in-patients in a country who has a 1.4-billion population. The recovered ICU patients had to wait a couple of days for a regular bed available to transfer in.

The other limitation is the self-made VAWCM-instillation package. The commercial VAC-Instill package is very costly. In China, most of the import medical instruments have to be paid by the patients themselves owing to the limited medical insurance coverage. In this study, the homemade VAWCM-instillation package, in which a mesh and nonadhesive plastic sheet were used to cover the exposed viscera, and suction tubes were placed between the mesh and the skin-attached drape. All the materials can be afforded. There are no technical problems with the self-made package. Nevertheless, that might increase the burden to both doctors and nurses.

In conclusion, the application of VAWCM-instillation resulted in an unimproved mortality but a significant increased delayed primary fascial closure rate in the patients with open septic abdomen.

## Figures and Tables

**Table 1:** Patients characteristics for final analysis.

	Pooled (*n* = 134)	Irrigation (*n* = 73)	Control (*n* = 61)	*P*
Age, years (range)	49 (20–79)	47 (20–79)	51 (28–77)	0.090
Gender (M : F)	92 : 42	52 : 21	40 : 21	0.482
Primary diagnosis, number (%)				
Postoperative anastomotic leakage without hemorrhage	51 (38.1)	26 (35.6)	25 (41.0)	0.524
Postoperative anastomotic leakage with hemorrhage	8 (6.0)	5 (6.8)	3 (4.9)	0.638
Severe acute pancreatitis	41 (30.6)	21 (28.8)	20 (32.8)	0.615
Perforation of gastric/duodenal/intestine	22 (16.4)	13 (17.8)	9 (14.8)	0.635
Complicated abdominal abscess	7 (5.2)	5 (6.8)	2 (3.3)	0.301
Other^a^	5 (3.7)	3 (4.1)	2 (3.3)	0.585
Classification of OA, number (%)				
Clean OA without adherence (1a)	37 (27.6)	21 (28.8)	14 (23.0)	0.445
Contaminated OA without adherence (1b)	59 (44.0)	34 (46.5)	25 (41.0)	0.471
Clean OA developing adherence (2a)	21 (15.7)	13 (17.9)	8 (13.1)	0.457
Contaminated OA developing adherence (2b)	2 (1.5)	1 (1.4)	1 (1.6)	0.705
OA complicated by fistula formation (3)	7 (5.2)	4 (5.5)	3 (4.9)	0.599
APACHE II score^b^, mean (range)	13.9 (7–29)	14.2 (9–28)	13.7 (7–29)	0.433

^a^Other diagnoses included complicated infected hematoma, septic incomplete abortion with traumatized uterus and perforation, acute ileus, and complicated cholecystitis. ^b^Acute physiology score and chronic health evaluation II.

The “1a, 1b, 2a, 2b, 3” are referred to the classification of the patients with septic OA (see [19]).

**Table 2:** The comparison of clinical outcomes between the irrigation and control groups.

Outcome	Pooled (*n* = 134)	Irrigation (*n* = 73)	Control (*n* = 61)	*P*
Mortality before abdominal closure^a^, *n* (%)	32 (23.9)	18 (24.6)	14 (23.0)	0.817
Primary fascial closure, *n* (%)	71 (53.0)	46 (63.0)	25 (41.0)	0.011
Primary fascial closure in the survivals^b^, *n*/total survivals (%)	71/102 (69.6)	46/55 (83.6)	25/47 (53.2)	0.001
Time to primary fascial closure, days (range)	25 (11–42)	23 (11–34)	28 (15–42)	0.003
Hospital LOS in the survivals^c^, days (range)	41 (19–88)	39 (19–88)	44 (27–79)	0.022
Hospital LOS in the survivals^c^ with fascial closure, days (range)	34 (19–53)	33 (19–44)	37 (27–53)	0.001
Complications				
Intra-abdominal abscess, *n* (%)	31 (23.1)	12 (16.4)	19 (31.1)	0.044
Postoperative fistula, *n* (%)	27 (20.1)	11 (15.1)	16 (26.2)	0.109
Postoperative hemorrhage, *n* (%)	11 (8.2)	7 (9.6)	4 (6.5)	0.524
Iatrogenic pneumonia, *n* (%)	24 (17.9)	10 (13.7)	14 (23.0)	0.164
Other (miscellaneous)^d^, *n* (%)	8 (6)	4 (5.5)	4 (6.6)	0.537

^a^Abdominal closure refers to the delayed primary fascial closure, partial fascial closure, skin grafting, or skin-only suturing. ^b^The survivals until abdominal closure. ^c^The survivals until hospital discharge. ^d^Other complications included catheter-associated infection, deep venous thrombosis, and pulmonary embolism.

**Table 3:** Factors related to fascial closure of the open septic abdomen.

Factors	Patients, *n* (%)	Fascial closure, events/total	OR	95% CI	*P *
Age, ≤60 y	99 (73.9)	55/71	1.484	0.684–3.219	0.316
Gender, male	92 (68.7)	50/71	1.19	0.573–2.472	0.640
Topical irrigation	73 (54.5)	46/71	2.453	1.222–4.927	0.011
Early enteral feeding	83 (61.9)	48/71	1.670	0.827–3.372	0.152
Early goal-directed fluid resuscitation	40 (29.8)	17/71	0.548	0.259–1.157	0.113
Restricted crystalloid fluid infusion	74 (55.2)	44/71	1.793	0.900–3.569	0.095
CRRT	48 (35.8)	26/71	1.077	0.530–2.186	0.838
